# iPSC-derived and Patient-Derived Organoids: Applications and challenges in scalability and reproducibility as pre-clinical models

**DOI:** 10.1016/j.crtox.2024.100197

**Published:** 2024-10-02

**Authors:** Elisa Heinzelmann, Francesco Piraino, Mariana Costa, Aline Roch, Maxim Norkin, Virginie Garnier, Krisztian Homicsko, Nathalie Brandenberg

**Affiliations:** a Doppl SA, Lausanne, Switzerland; b Department of Oncology, CHUV, Lausanne, Switzerland

**Keywords:** Induced Pluripotent Stem Cells **(**iPSCs), 3D cell culture, Organoids, Patient-Derived Organoids (PDOs), Micro Physiological Systems (MPS), Pre-clinical models

## Abstract

Recent advancements in stem cell technology have led to the development of organoids – three-dimensional (3D) cell cultures that closely mimic the structural and functional characteristics of human organs. These organoids represent a significant improvement over traditional two-dimensional (2D) cell cultures by preserving native tissue architecture and cellular interactions critical for physiological relevance.

This review provides a comprehensive comparison between two main types of organoids: induced Pluripotent Stem Cell (iPSC)-derived and Adult Stem Cell (ASC)-derived (also known as Patient-Derived Organoids, PDOs). iPSC-derived organoids, derived from reprogrammed cells, exhibit remarkable plasticity, and can model a wide range of tissues and developmental stages. They are particularly valuable for studying early human development, genetic disorders, and complex diseases. However, challenges such as prolonged differentiation protocols and variability in maturation levels remain significant hurdles. In contrast, ASC-derived organoids, generated directly from patient tissues, faithfully recapitulate tissue-specific characteristics and disease phenotypes. This fidelity makes them indispensable for personalized medicine applications, including drug screening, disease modeling, and understanding individualized treatment responses.

The review highlights the unique advantages and limitations of each organoid type, emphasizing their roles in advancing biomedical research and drug discovery. It addresses key challenges in organoid technology, such as scalability, reproducibility, and the need for standardized culture protocols. Furthermore, it explores recent innovations in scaffold-guided organoid engineering and the integration of organoids with advanced technologies like artificial intelligence and high-throughput screening.

The integration of organoids with cutting-edge technologies holds promise for enhancing their utility in modeling complex human diseases and accelerating drug discovery and development. By providing more physiologically relevant models of human organs, organoid technology is poised to revolutionize biomedical research, offering new insights into disease mechanisms and personalized therapeutic strategies.

## Highlights

• Organoids mimic *in vivo* human organs, surpassing 2D cultures in complexity.
• Pluripotent Stem Cell (PSC)-derived organoids can model developmental stages.
• Patient-derived organoids (PDOs) reflect patient-specific disease states.
• Organoids aid in drug discovery, improving efficacy and toxicity studies.
• Future work focuses on scalable and biomimetic methods.

## Graphical abstract

**Figure:**
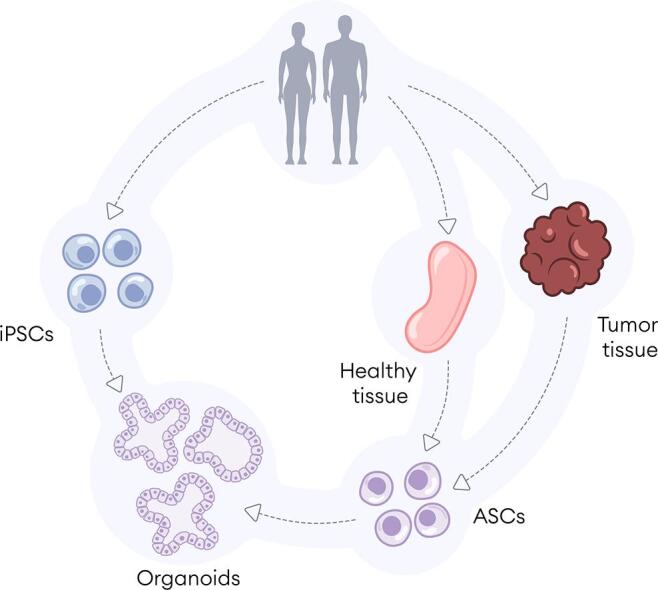

## Introduction

Stem cell technology has evolved significantly over the years, providing the ability to differentiate cells into complex structures that closely mimic *in vivo* organs. Historically, stem cell-based research was conducted using two-dimensional (2D) culture systems. However, cells in 2D cultures fail to replicate the normal morphology and interactions observed *in vivo*. When cultured in a 2D environment, isolated tissue cells gradually lose their shape, flatten, and divide abnormally, impacting their differentiation and function (Baker and Chen, 2012, Saraswathibhatla et al., 2023). The 2D attachment of cells in these conditions leads to a loss of structural organization and affects cell–cell and cell-extracellular matrix (ECM) interactions, resulting in cellular phenotypes that do not accurately reproduce the functions and behaviors of tissues or organs (Scalise et al., 2021). Additionally, 2D models derived from tumor cells, tend to lose their heterogeneity over long-term cultures, with their genomic and metabolic profiles diverging significantly from the original tumors (Bresnahan et al., 2020).

To address these limitations, organoids have emerged as a significant advancement in stem cell research over the past decade. Organoids are three-dimensional (3D) cell cultures derived from stem cells that have the capability to self-organize and differentiate into structures resembling their corresponding organs (Corrò et al., 2020). The development of organoids was initiated with the successful culture of intestinal adult stem cells (ASCs) in 2009 by Toshiro Sato and his colleagues, forming small intestinal organoids with a crypt-villi structure (Sato et al., 2009). Since then, various organoid protocols have been established to model the wide range of human organs. These 3D model systems more accurately replicate the *in vivo* microenvironment and interactions with various cell types. These cellular systems maintain their complex structures and specific functions, preserving genetic stability and cellular heterogeneity (Yang et al., 2023).

Organoids have become invaluable tools in various fields such as developmental biology, disease modeling, and drug discovery (Lehmann et al., 2019, Silva-Pedrosa et al., 2023, Done and Birkeland, 2023, Piraino et al., 2024). They offer a robust platform for studying disease mechanisms and testing potential therapies. Organoids are extensively used in drug discovery and development, enabling high-throughput screening of potential therapeutics, and predicting drug responses. The use of organoids in preclinical studies enhances the accuracy of drug efficacy and toxicity assessments, ultimately improving the translation of findings from the laboratory to clinical settings (Yang et al., 2023). Their application also extends to personalized medicine, where patient-derived organoids can be used to tailor treatments to individual genetic and phenotypic profiles, potentially leading to more effective and targeted therapies (Li et al., 2020). Tumor organoids, derived from biopsies or tumor resections, play a crucial role in personalized medicine by predicting drug sensitivity for individual patients (Clevers, 2016). Thus, organoids provide enhanced options for drug screening and personalized therapeutic approaches.

While the variety of organoid models continues to expand, the cellular sources for organoid generation primarily converge on two main types: induced pluripotent stem cell (iPSC)-derived organoids and adult stem cell (ASC)-derived organoid (Hofer and Lutolf, 2021). iPSC-derived organoids are generated from reprogrammed iPSCs, which can differentiate into various cell types representing different organ systems (Rowe and Daley, 2019). Due to this plasticity, iPSC-derived organoids are used to model a wide range of tissues and developmental stages, making them particularly useful for studying early developmental processes, disease mechanisms, and genetic disorders. In contrast, ASC-derived organoids or patient-derived organoids (PDO) are generated by directly dissociating healthy or diseased tissues and then culturing them under conditions with tissue-specific growth factors (Kim et al., 2024). Therefore, ASC-derived organoids recapitulate the original tissue phenotypes more consistently, making them exceptionally valuable for personalized medicine as they accurately reflect the patient's specific disease state.

In this review, we compare iPSC-derived organoids and ASC-derived organoids, focusing on their unique characteristics, applications, and challenges in biomedical research. Through this comprehensive comparison, we aim to guide researchers in selecting the most suitable organoid models for their specific preclinical studies and research objectives.

By examining the strengths and limitations of each organoid type, this review provides insights into their roles in advancing our understanding of human biology and disease. Additionally, we will explore general challenges in organoid research, such as scalability and highlight recent trends and future perspectives in 3D cell engineering and its applications, highlighting how these advancements can further enhance the utility and precision of organoid models in biomedical research.

## iPSC-derived organoids

In 2006 and 2007, Yamanaka and colleagues made the pioneering discovery that terminally differentiated cells can be reprogrammed into pluripotent stem cells (PSCs). This pivotal moment in scientific research opened a new field in stem cell studies (Takahashi et al., 2007, Takahashi and Yamanaka, 2006). Human induced pluripotent stem cells (hiPSCs) exhibit striking similarities to human embryonic stem cells (hESCs) in their genetic and epigenetic characteristics, as well as their capacity for multilineage differentiation (Narsinh et al., 2011). However, iPSCs offer distinct advantages over hESCs, especially in their ease of derivation from individual healthy and diseased donors, thereby circumventing ethical concerns inherent to embryonic sources. Consequently, 2D iPSC-based models have emerged as a cornerstone in biomedical research. The generation and characterization of hiPSCs is a well-established process. While the costs of iPSC generation and validation are high, iPSCs banks were founded worldwide, to collect and generate hiPSCs for scientific research (Huang et al., 2019). Despite their advantages of representing simple and economically attractive models, they also come with inherent limitations. The simplicity of 2D culture fails to capture the intricacies of human organ architecture, cellular heterogeneity, and structural features. Moreover, they lack crucial cell–cell and cell-extracellular interactions essential for mimicking physiological conditions accurately.

**Fig. 1:**
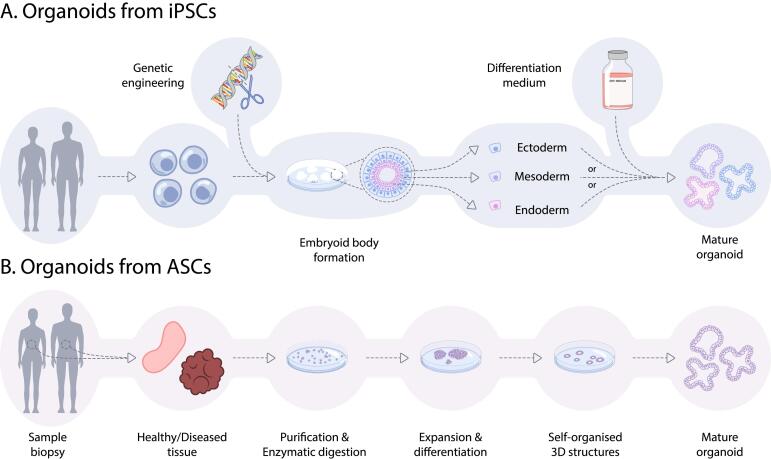
**Organoids generation from iPSCs and ASCs. A:** iPSC-derived organoids originate from 2D cultures to be grown into embryoid bodies (EBs). At this point, they may be embedded in an extracellular matrix. EBs are expanded and differentiated into the germ layer lineage of interest by the addition of tissue-specific growth factors. Final organoid maturation is achieved by using a growth factor rich media that is specific to the tissue of interest. Additionally, iPSCs can be genetically engineered on their way of differentiation into mature organoids.** B:** ASC-derived organoids are grown from healthy or tumor tissue biopsies. Tissues are processed into a single cell or small fragment suspension which is directly embedded in an extracellular matrix. Media containing numerous tissue-specific growth factors, is added, and regularly changed until organoids have expanded.

In recent years, iPSC-derived organoids have emerged as a transformative stem-cell-based 3D model, addressing the constraints of traditional 2D models, and circumventing the limitations of human primary tissues availability, from which ASC organoids are derived. Organoids that are cultivated from iPSCs, harness the remarkable capacity of iPSCs to self-organize into small, unstructured aggregates, so called embryoid bodies (EBs) (Sahu and Sharan, 2020). Depending on the tissue and organ of interest, self-assembled iPSCs can be directed by growth factors and small molecules to induce the differentiation into the three different germ layer lineages, the ectodermal, the mesodermal or the endodermal lineage (Fig. 1). Organs originating from the endoderm layer encompass complex organ systems, such as the gastrointestinal and respiratory tract. Organs derived from the mesodermal lineage include the kidney, muscles, heart, and blood vessels. The ectodermal lineage is associated with the nervous system, including the brain, eyes, ears, and more.

**Fig. 2:**
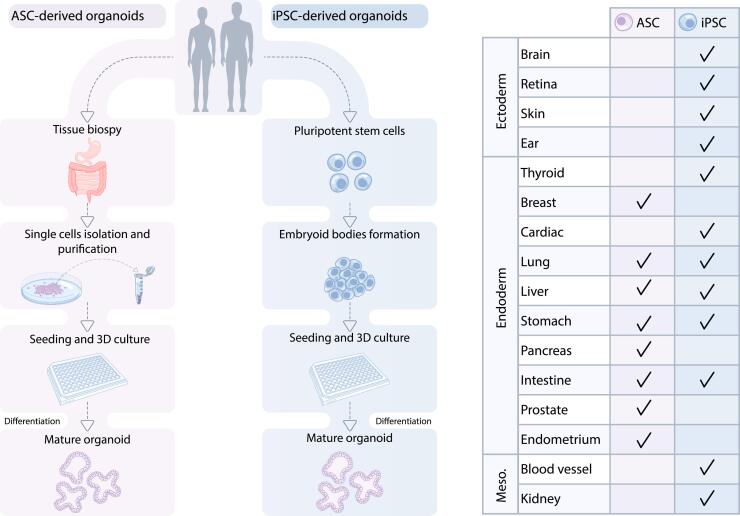
**ASCs and iPSCs-derived organoid culture steps and overview of human organoid models and their respective origins.** To generate organoids from ASC, tissue samples are obtained from human medical interventions. The tissue samples are opened, washed, and then cut into small fragments (2–4 mm) to increase the surface area for enzymatic digestion or further mechanical dissociation to isolate single stem cells. After several rounds of washing and purification, the harvested stem cells will be used for seeding and generation of organoid cultures for expansion. To generate organoids from iPSC using genetic engineering, iPSCs are maintained and expanded as undifferentiated clonal populations on feeder cells or defined extracellular matrix (ECM) substrates to aggregate to embryoid bodies. Typically, iPSCs are harvested as cell aggregates, which preserve cell–cell contact and yield cell populations with higher viability. These aggregates are further induced through germ layer specification to form mesodermal domes, endodermal spheres, and ectodermal matrix for additional applications. The illustrated chart provides information on the type of existing human organoid models and their respective origins (i.e. ASC- or iPSC-derived).

Interestingly, only a small number of pathways are involved in directing the germ layer formation, such as the Wnt, FGF, retinoic acid (RA) and TGFβ/BMP pathways (McCauley and Wells, 2017). Through different combinations of small molecules and growth factors, these pathways can be regulated to generate human organoids that resemble various tissues and organoids, such as the brain (Lancaster et al., 2013), eyes (Nakano et al., 2012), kidney (Takasato et al., 2014), lung (Dye et al., 2015), gastric tissue (McCracken et al., 2014) and intestine (Spence et al., 2011) have been already successfully generated (Fig. 2).

Notably, iPSC-derived organoids have gained increasing attention by faithfully recapitulating tissues derived from the ectodermal lineages, thus effectively modeling intricate organs such as the brain, eye, and inner ear. Access to these tissues is exceptionally restricted, and generating such organoids from adult tissue-derived stem cells has not yet been achieved. Another prominent example of iPSC-derived organoids are kidney organoids, originating from the mesodermal lineage. iPSC-derived kidney organoids resemble the first trimester of the human fetal kidney tissue and mimic the complex kidney structure consisting of nephrons and the collecting duct network (Takasato et al., 2014).

The following sections will explore the generation process of iPSC-derived organoids, focusing specifically on brain organoids. We will also discuss the applications of iPSC-derived organoids, their inherent limitations, and the challenges they present in biomedical research.

### Generation of iPSC-derived organoids

In general, protocols for the generation of iPSC-derived organoids are more complex and time consuming compared to ASC-derived organoids. This is mainly due to the fact that iPSCs have a pluripotent character and must be first guided into the germ layer of interest to finally initialize their differentiation into the desired tissue or organ. In contrast, ASCs are already committed to organ-specific differentiation.

IPSCs are cultivated and expanded over numerous generations under naïve cell culture conditions. To preserve their undifferentiated state, iPSCs are typically cultured on feeder cells or extracellular matrix (ECM) coated surfaces. Within these culture conditions, iPSCs proliferate and form clonal populations. Prior to seeding for organoid generation, iPSCs must undergo mechanical detachment and enzymatic dissociation (Beers et al., 2012). The generation of organoids derived from iPSCs relies on their inherent “self-aggregation” capability (Xie et al., 2017). To facilitate this self-aggregation into EBs, cells can be cultured in round-, U- or V-bottomed multi-well plates or using rotational forces thus accelerating their aggregation (Nie and Hashino, 2020) (Fig. 2). Additionally, microfluidic systems have emerged as a valuable tool for the continuous generation of cell aggregates, particularly beneficial for high-throughput applications (Yu et al., 2019).

During their cultivation and differentiation, organoids can be maintained in a scaffold-free environment, embedded within an extracellular matrix (ECM) gel (Aisenbrey and Murphy, 2020), or structured into a three-dimensional (3D) architecture using external biomaterial scaffolds (Jeon et al., 2022). These approaches offer flexibility in tailoring the organoid's microenvironment to support its maturation and functionality. In general, iPSC organoids can be cultured and passaged for a long time maintaining a stable genotype.

A critical phase in iPSC-derived organoid formation is the commitment of cells to the required embryonic germ layers, ectoderm, mesoderm, or endoderm (Kiecker et al., 2016) (Fig. 1A). This commitment is orchestrated by the application of specific differentiation-inducing factors and pathways, such as the wingless-type mouse mammary tumor virus integration site family (WNT), transforming growth factor beta (TGF-β) and fibroblast growth factor (FGF) signaling pathways (Lewandowski et al., 2017). In contrast, ASCs inherently possess a certain degree of lineage commitment, making the initial germ-layer differentiation step unnecessary when using ASCs as starting material (Fig. 1B). Upon commitment to a germ layer, tissue-specific growth factors play a pivotal role in directing these differentiated cells towards adopting the characteristics of the target tissue or organ. Acting as signaling molecules, these growth factors further fine-tune cellular fate, ensuring the development of the desired tissue morphology and functionality. For example, iPSC-derived retinal organoids can be generated by activating either the BMP4 or the IGF1 signaling pathways, depending on the iPSC cell line. BMP4 plays a role in directing the anterior portion of the neural plate towards retinal neurons, while IGF1 promotes retinal fate induction. For long term maturation of retinal organoids, other components such as retinoic acid (RA), taurine, and triiodothyronine (T3) can be added to the culture medium (Chichagova et al., 2020).

In conclusion, the complex nature of iPSC-derived organoid generation, involving multiple steps to guide pluripotent cells into specific germ layers and subsequent differentiation into desired tissues or organs, contrasts with the relatively simple generation of ASC-derived organoids (1). While iPSCs require meticulous cultivation and differentiation protocols, including the use of specific signaling pathways and growth factors, ASCs inherently possess lineage commitment, streamlining the process.

### Applications of iPSC-derived organoids

**Fig. 3:**
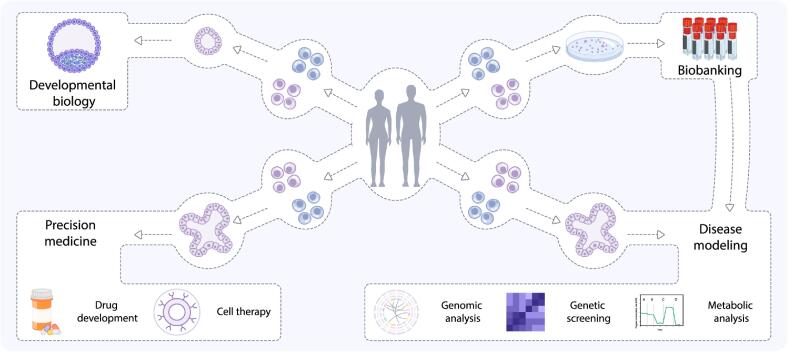
**Applications of organoids.** A schematic summary of the various applications of organoids, including developmental biology, biobanking, disease modeling (genomic analysis, genetic engineering, metabolic analysis), and precision medicine (drug development, cell therapy).

To date, organoids have emerged as versatile tools with multifaceted applications in biomedical research, drug discovery and medicine. They serve as models for diseases, offering insights into pathological mechanisms. Furthermore, organoids serve as robust platforms for high-throughput drug screening, facilitating the identification of novel therapeutics. IPSC-derived organoids in particular serve as tools for understanding the processes underlying human development and diseases. The integration of iPSC-derived organoids with genome editing methodologies extends their utility, enabling precise manipulation and interrogation of disease-related pathways (Fig. 3). More recently, iPSCs have also emerged as a promising cell replacement therapy for tissue regeneration of currently uncurable degenerative diseases, such as Parkinson’s disease (Schweitzer et al., 2020).

#### iPSC-derived organoids to study human development

Understanding human-specific developmental mechanisms requires suitable human model systems. iPSC-derived organoids excel in this regard, as they can develop into organ-like structures from all three germ layers, faithfully mirroring early embryonic development towards fetal stages. This ability allows for the exploration of developmental phases previously inaccessible with other models (Fig. 3).

Since the discovery of pluripotent stem cell lines, scientists have aimed to understand embryonic development and organ formation by directing naïve cell differentiation into various post-mitotic cell types. A significant breakthrough occurred in 2013 when Yoshiki Sasai and his team demonstrated that both mouse and human embryonic stem cells can develop into highly complex structures resembling normal tissues and organs when cultured in 3D environments (Sasai, 2013). Recent studies have revealed that ESCs and iPSCs can also self-organize into embryo-like structures, such as blastoids and gastruloids, which accurately mimic early embryonic development. Blastoids, which model the blastocyst stage (pre-implantation), can be generated by combining mouse ESCs and extra-embryonic trophoblast stem cells (TSCs) (Rivron et al., 2018). Gastruloids model the gastrulation stage (post-implantation) and can be generated by self-organizing mouse ESCs in the presence of Wnt inhibitors and constant whisking. They can replicate key stages of embryo development, including the formation of different cell layers and body axes (Beccari et al., 2018). The development of embryo-like structures and organoids is still in its early stages, and neither model can sustain de novo embryonic development beyond a few days (Li et al., 2019). Nevertheless, blastoids and gastruloids have opened new avenues to understand embryogenesis, more particularly in humans, with wide application in biomedical discoveries, such as the possibility to generate organ structures inside embryo models and the better understanding of developmental malformations.

Similarly, iPSC-derived organoids have become a powerful tool for studying human brain development, offering insights into the complex processes of neurogenesis and brain organization (Lancaster et al., 2013). Brain organoids mimic key features of the developing human brain, including cellular diversity, structural organization, and functional neural networks. Their ability to replicate aspects of human brain physiology and pathology positions brain organoids as invaluable tools for studying neurodevelopmental disorders, neurodegenerative diseases, and infectious diseases affecting the brain.

The number of published protocols for generating brain organoids continues to grow, along with the diversity of distinct brain organoid types. Broadly, there are two methods for generating brain organoids: unguided and guided (Hopkins et al., 2021). In the unguided method, hiPSC aggregates undergo spontaneous morphogenesis and intrinsic differentiation, leading to the formation of cerebral organoids. These organoids encompass a broad spectrum of cell lineage identities, including forebrain, midbrain, and hindbrain regions, as well as retinal, choroid plexus, and mesodermal tissues (Lancaster et al., 2013, Camp et al., 2015). Large-scale single-cell transcriptomic profiling has revealed the extensive cellular diversity within cerebral organoids. These organoids encompass the majority of cell types found in the native central nervous system (CNS), including neural progenitors, excitatory and inhibitory neurons, astrocytes, and oligodendrocyte precursor cells (OPCs) (Quadrato et al., 2017). Consequently, cerebral organoids closely mimic the cellular composition and organization of the developing human brain, providing a valuable model for studying neurodevelopmental processes. The guided method requires the supplementation of the culture medium with small molecules and growth factors to induce differentiation toward specific brain regions, such as the cerebral cortex, forebrain, hippocampus, and midbrain (Camp et al., 2015, Quadrato et al., 2017, Qian et al., 2016, Bagley et al., 2017). This approach allows for more targeted studies of particular areas of the brain and their associated functions and pathologies. For example, forebrain organoids, generated and cultured in multi-well spinning bioreactors, consistently form cortical structures with distinct layers, resembling the ventricular zone (VZ), inner and outer subventricular zones (SVZ), and the cortical plate (CP) (Qian et al., 2016). The enlarged outer subventricular zone in these forebrain organoids provides opportunities for investigating human cortical development and related disorders.

To better model interactions between different brain regions, researchers have developed new methods where hiPSCs are first directed to a specific brain region organoid separately. These region-specific organoids are then fused to create complex structures, also called “assembloids”, with multiple distinct regional identities in a controlled manner. For example, by fusing dorsal and ventral forebrain organoids, interneurons from the ventral domain migrate towards the dorsal domain, mimicking the natural migration pattern observed *in vivo*, where interneurons move from the subpallium to the cerebral cortex (Bagley et al., 2017).

Taken together, these advancements highlight the significant potential of iPSC-derived organoids in modeling human development. They provide a powerful platform for studying complex biological processes, such as development of the human brain, paving the way for new discoveries in developmental biology and precision medicine. By continually refining these models, scientists will be better equipped to investigate the intricacies of human development.

#### iPSC-derived organoids to model diseases

Beyond their advantages for developmental studies, iPSC-derived organoids are extensively utilized to investigate human diseases (Fig. 3). These organoids can be generated from patient-specific iPSCs, allowing researchers to study the impact of disease-related genetic mutations on organ development and function. This approach enables the creation of highly personalized disease models, facilitating the exploration of pathogenesis and the testing of potential therapeutic interventions.

In cancer research, iPSC-derived organoids have been used to model tumor development stages for various types of cancers, particularly hereditary cancers with germline oncogenic mutations. An important benefit of using iPSCs in cancer studies is their ability to generate cell lines tailored to specific diseases. By reprogramming cells obtained from cancer patients, iPSCs can be cultivated to harbor the same genetic abnormalities observed in the patient's tumor cells (Maruoka et al., 2022). For instance, kidney cancer organoids could be derived from iPSC lines from patients with hereditary c-met-mutated papillary renal cell carcinoma (PRCC) (Hwang et al., 2019). The generated organoid model exhibits typical markers present in primary tumors of c-met-mutated PRCC. These models can be used to screen for drug sensitivity, helping tailor personalized treatment plans. This personalized approach is crucial for developing effective cancer therapies, as it accounts for the genetic diversity and heterogeneity of tumors.

In autism research, iPSC-derived brain organoids from patients with autism spectrum disorder (ASD) have revealed insights into the neurodevelopmental abnormalities associated with the condition, such as altered synaptic connectivity and neuronal differentiation (de Santos et al., 2023). Similarly, in Alzheimer's disease, brain organoids derived from patients' iPSCs have been used to model amyloid plaque formation and tau pathology, providing a platform for testing potential therapeutic agents (Marei et al., 2023).

Overall, iPSC-derived organoids represent a powerful and versatile tool for disease modeling, providing a human-relevant platform for studying the mechanisms and onsets of disease, identifying potential biomarkers, and testing new therapeutic interventions. Their ability to replicate patient-specific disease phenotypes makes them particularly valuable for precision medicine.

#### Genome engineering in iPSC-derived organoids

A distinctive advantage of iPSCs is their compatibility with genome editing techniques. The combination of iPSC-derived organoids and gene-editing technologies has led to the generation of numerous disease models with great potential in the field of precision medicine. Genome editing can be used to induce specific changes in an otherwise identical genetic iPSC background. The CRSPR/Cas9 system has been established as the major tool of genome editing in recent years (Go and Stottmann, 2016). This targeted nuclease-based technology enables the accurate manipulation of genomic sequences, and both, gain- and loss-of-function phenotype disease models can be created by the CRISPR/Cas9 technology (Grajcarek et al., 2019). Like this, isogenic pairs of disease-specific organoids and control organoids can be generated. This approach allows researchers to investigate the specific role of a mutation within the same genetic background. At the same time, disease-causing mutations in patient-derived iPSCs can be corrected (Jang and Ye, 2016).

Genome editing in iPSC-derived organoids can be conducted either at the pluripotent stage, prior to their differentiation into the desired cell or organoid type, or at various points along the differentiation process.

However, it must be noted, that the delivery of genome-editing agents into 3D organoids is more difficult than in simple 2D cell lines. The editing efficiency in 2D can reach up to 95 %, whereas vector-based delivery approaches in 3D organoids can be as low as 10–30 % (Geurts and Clevers, 2023, Fujii et al., 2015). Newer methods, such as the ribonucleoprotein (RNP)-based CRISPR approach can also result in 3D editing efficiencies higher than 90 % (Kim et al., 2014, Skoufou-Papoutsaki et al., 2023).

One example of genome engineering involves lung organoids. HiPSC-derived lung progenitor cells were used to generate alveolar organoids that include functioning type 2 alveolar epithelial cells with lamellar bodies, which secrete surface-active proteins. In iPSCs from patients lacking surface-active protein B (SFTPB), the derived alveolar organoids are missing their lamellar bodies within the type 2 alveolar epithelial cells and cannot produce SFTPB (Jacob et al., 2017). These findings suggested that correcting SFTPB mutations through gene editing could rescue the phenotype of specific patient-derived iPSCs. Consequently, hiPSC-derived lung organoids hold significant promise for applications in disease modeling and drug screening for lung diseases.

Another powerful approach involving gene editing in iPSCs and organoids is the use of reporter genes to trace cell fate during the development or the progression of specific diseases. By constructing knock-in reporter genes for specific target genes, researchers can visualize and monitor the behavior, differentiation, and lineage of cells in real-time (Zhou et al., 2021). For instance, fluorescent reporters can be inserted into the genome at precise locations. When these genes are expressed, the fluorescent signal allows researchers to track where and when specific cells differentiate, migrate, or undergo other crucial processes (Jung et al., 2017). These reporter systems can be combined with organoid disease models. For example, tumor suppressors and oncogene mutations can be introduced together with GFP into healthy cerebral forebrain organoids by CRISPR/Cas9. This allows to monitor clonal, tumorigenic-like outgrowth of genetically engineered fluorescently labeled cells in the context of a normal cerebral forebrain organoid (Schönrock et al., 2022).

In conclusion, the combination of iPSC-derived organoids and genome editing technologies marks a significant advancement in biomedical research and allows for the creation of accurate disease models and the correction of genetic mutations, providing a powerful platform for drug discovery and personalized treatments.

### Challenges and limitations of iPSC-derived organoids

#### Regulatory compliance and ethical considerations

To fully harness the potential of iPSC organoid technology in biomedical research, precision medicine, and disease modeling, numerous challenges and limitations must be addressed. One of the most significant challenges in this context is the development and establishment of clinical grade iPSCs under strict ethical and legal policies.

Ensuring that all iPSCs meet the strict regulatory requirements for clinical use and safety is a primary concern. For instance, early reports on iPSC generation highlighted tumor formation in more than 20 % of the iPSCs due to the reactivation and overexpression of the c-Myc oncogene (Okita et al., 2007, Ahmed et al., 2011). To minimize these risks, regulatory authorities set high standards to ensure that iPSC-derived products are safe and effective. This includes comprehensive testing for genetic stability, absence of tumorigenicity, and consistent quality of the derived cells.

In addition to regulatory compliance, ethical considerations are crucial for maintaining public trust and adherence to legal standards. This involves obtaining informed consent from donors, navigating intellectual property issues, ensuring the ethical use of genetic materials, and addressing potential privacy concerns related to genetic information (Lo and Parham, 2009, Omole et al., 2022).

#### Scientific and practical limitations of iPSCs technology

Besides the discussed legal and ethical limitations, the generation, standardization, handling and maintenance of iPSCs also involves practical, scientific challenges. There is a persistent need for standardized protocols for iPSC generation and differentiation. Variability in methods can lead to inconsistencies in cell quality and behavior, reducing reproducibility and reliability across different laboratories and studies. Standardization ensures uniformity in research and clinical applications and facilitates more consistent and comparable results. Implementing robust quality control measures is essential to ensure the reproducibility and safety of iPSCs. This includes regular screening for genetic abnormalities and contaminations, as well as functional assays to verify the pluripotency and differentiation potential of the cells (Assou et al., 2020, Liu and Zheng, 2019). Ensuring the long-term viability and genetic stability of stored iPSC lines is also critical for maintaining a reliable iPSC biobank. This involves optimal cryopreservation techniques and regular monitoring of stored cells to prevent genetic drift and degradation over time.

Another important consideration is to ensure that the biobank includes iPSCs from diverse ethnic, genetic and gender backgrounds. This is especially important when conducting research on diseases that affect different populations (Chehelgerdi et al., 2023). This diversity is crucial for developing therapies that are effective across various demographic groups.

Addressing these challenges and fulfilling all the necessary requirements in the generation and maintenance of clinical grade iPSCs is labor-intensive and costly, as specialized facilities, equipment, and skilled personnel are needed. These expenses can be prohibitive, particularly for smaller research institutions and companies. Therefore, reducing costs through technological advancements and optimized protocols is crucial for making iPSC technologies more accessible.

Overcoming these limitations is vital for the successful implementation of iPSC organoid technology in clinical research, precision medicine and disease modeling.

#### Limitations in applications of iPSC-derived organoids

One major issue of the application of iPSC-derived organoids to biomedical research and clinical settings is the immature functional characteristics exhibited by many iPSC-derived cells, which often mirror embryonic or fetal developmental stages and lack the mature functional traits required for accurate disease modeling and therapeutic testing. Efforts to promote maturation in iPSC-derived cells have explored various strategies. In the case of maturation of iPSC-derived cardiomyocytes, this could include for example prolonged culture times, supplementation with thyroid hormone or extracellular matrices (Lewandowski et al., 2018, Yang et al., 2014, Thavandiran et al., 2013). However, these approaches have shown limited success in achieving the fully mature functional properties needed for effective clinical applications.

Another drawback of iPSC-derived organoids is that they might not always represent the region of the organ investigated. For example, iPSC-derived intestinal organoid protocols spontaneously drive differentiation towards small intestinal organoids instead of other intestinal regions, such as the colon or cecum. However, with increasing knowledge on colonic tissue development in the recent years, the characterization of specific modulators of colonic signaling pathways, such as BMP, has allowed the development of iPSC-derived organoids specifically into colonic tissue (Takahashi et al., 2017, Múnera et al., 2017). The need of multiple growth factors and signaling molecules during iPSC organoid generation also shows that these protocols are more complex and time consuming compared to the generation of organoids derived from adult, patient-derived stem cells.

Addressing these challenges by developing more effective maturation techniques and refining experimental protocols are essential steps toward overcoming these limitations and maximizing the clinical utility of iPSC-derived phenotypic cells.

## Patient-derived organoids (PDOs)

The rapid evolution of 3D culture technologies has significantly enhanced the development of physiological human tissue models *in vitro*, transforming our ability to study cancer and other disease progression. These advancements have led to the generation of patient-derived organoids (PDOs), which are self-organizing, miniature versions of organs derived from tissue-specific ASCs (Fig. 1). PDOs closely mimic the architecture and functionality of human organs and provide a valuable model system for biomedical research, drug screening, and clinical applications (Yang and Yu, 2023).

### Generation of patient-derived organoids

PDOs are typically derived from healthy tissue biopsies by embedding extracted ASCs into a 3D extracellular matrix such as Matrigel, which provides the necessary extracellular mechanical and biochemical support. Cells are then grown in specialized media formulations supplemented with tissue-specific growth factors that can simulate their *in vivo* stem cell niche *in vitro*.

In 2009, Sato et al. demonstrated that mouse epithelial organoids could be generated from a single sorted leucine-rich repeat-containing G-protein-coupled receptor 5 (LGR5)+ intestinal stem cell in the presence of three essential factors like R-spondin 1 (a WNT agonist), epidermal growth factor (EGF), and the bone morphogenetic protein (BMP) inhibitor Noggin (Sato et al., 2009). These conditions allow extracted LGR5+ cells to form highly polarized epithelial structures with distinct proliferative crypts and differentiated villus compartments. Indeed, both proliferative and differentiated cells were represented in the resulting organoids, including Paneth cells, goblet cells, and enteroendocrine cells, among others, at normal ratios comparable to those *in vivo* (Sato et al., 2009). Importantly, these protocols have been well-established and successfully reproduced in numerous studies (Brandenberg et al., 2020).

The successful culture of these organoids laid the groundwork for establishing organoids from mouse and human epithelial intestinal tissues, as well as from other gastrointestinal tract tissues and various other organs such as the lung and breast (Mitrofanova et al., 2023, Barkauskas et al., 2017, Sachs et al., 2018).

Indeed, human intestinal organoids were shown to require a distinct cocktail of growth factors compared to mice, such as the need for additional exogenous Wnt supplementation. Moreover, replacing p38i and EGF with insulin-like growth factor 1 (IGF-1) and fibroblast growth factor 2 (FGF-2), along with stable Wnt alternatives, improved the control and maturation of human intestinal organoids, leading to more physiological crypt-like structures and diverse intestinal cell types (Miao et al., 2020).

Growing organoids from human organs frequently targeted by drug-induced toxicities (gut, liver, kidney) can complement or even replace animal-based toxicology with human tissue assays. For instance, intestinal organoids can be used to study drug and chemotherapy side effects like nausea, vomiting, and diarrhea (Peters et al., 2019, Peters et al., 2020).

The ability to generate organoids from healthy tissues has paved the way for creating organoids from patient-derived tumor tissues. To generate tumor organoids, biopsies from primary or metastatic tumor sites are collected and processed using standard protocols (Driehuis et al., 2020). The tissue is often dissociated into single cells or small fragments and then embedded in ECM. The culture medium for tumor organoids typically contains components that support cancer cell growth while suppressing the growth of normal cells. As an example, in colorectal cancer (CRC) organoids, which often have activating mutations in the WNT signaling pathway, a medium lacking WNT and R-spondin 1 can be used to selectively culture cancer cells. Similarly, for tumors with mutations in the EGF receptor (EGFR) signaling pathway, EGF withdrawal can be used to suppress the growth of normal cells (Sato et al., 2009, Sato et al., 2011).

Given the unknown genomic background of tumors at the time of biopsy, it is essential to test several culture media to capture the full spectrum of tumor organoid formation. Different combinations of growth factors and inhibitors tailored to the specific requirements of various tumor types and their genetic mutations ensure the efficient establishment of physiologically relevant organoids.

Various studies demonstrated successful long-term culture of organoids derived from primary colon, esophagus, pancreas, and well as prostate and ovarian cancer. These tumor-derived organoids can maintain the genetic and phenotypic characteristics of the original tumors, making them excellent models for studying cancer biology and drug screening as reviewed by Driehuis and colleagues (Driehuis et al., 2020).

A major challenge in tumor organoid generation is the overgrowth of normal organoids, which can be mitigated by using pure tumor material or selective culture conditions favoring cancer cells. Identifying organoid morphology can be beneficial, as normal organoids present a single-layered, cyst-like structure, while tumor organoids mimic the glandular, solid, and poorly cohesive structures of their original cancer tissues. Interestingly, tumor organoids do not grow faster than their normal counterparts and often grow at slower, which can also hinder tumor organoid generation success.

Numerous studies have showcased the potential of organoid technology in cancer research. The establishment of biobanks containing large collections of patient-derived tumor organoids and matching healthy organoids is a significant advancement in cancer research. These biobanks provide a valuable resource for studying cancer biology, identifying biomarkers, and testing drug responses. Recently, biobanks of tumor organoids from CRC, PDAC, breast, and ovarian cancers, amongst several others have been established, including genetically diverse tumor and normal tissue-derived organoids.

Colorectal cancer organoids biobanks were one of the first to ever been reported. In 2015, a CRC organoid biobank was established, demonstrating that the organoids accurately recapitulate the genetic and phenotypic features of the original tumors. They performed a high-throughput drug screen, showing that the drug responses of CRC organoids were well correlated with clinical outcomes (van de Wetering et al., 2015).

Following a similar approach, Herpers et al. used a CRC and healthy organoid biobank to identify MCLA-158, a dual-targeting antibody for WNT signaling and EGFR, which effectively inhibited CRC organoid growth with minimal toxicity to benign LGR5+ stem cells using an image base high-content screening approach (Herpers et al., 2022). Large organoid biobanks continued to be established for several other cancer types and in 2018, a study generated organoids from 138 patients with pancreatic cancer, revealing genetic and transcriptomic signatures linked to drug responses that mirrored clinical outcomes, and highlighting the potential of organoids for predicting treatment responses (Tiriac et al., 2018).

Sachs et al. (2018) also created a biobank of breast cancer organoids from over 100 patients, retaining original tumor features, including hormone receptor status. A few organoids lost receptor status, highlighting the need for thorough characterization. A drug screen targeting HER2 signaling showed sensitivity correlated with HER2 status (Sachs et al., 2018).

Despite their advantages, organoid biobanks face challenges like variability in culture conditions and the need for standardized protocols. Developing synthetic or biomimetic matrices to replace Matrigel is one of the crucial steps for achieving reproducibility standards that meet the pharmaceutical and healthcare industry needs for large scale adoption. Future research should refine culture methods, explore co-cultures with stromal and immune cells, and expand biobanks to cover more cancer types and genetic profiles.

Overcoming these challenges will motivate the broader use of organoid biobanks in various applications. Organoid biobanks offer immense potential in personalized medicine, drug screening, and understanding cancer biology and resistance. By accurately reflecting the genetic background and heterogeneity of original tumors, these biobanks will enable researchers to test therapies and study cancer progression in a controlled, reproducible environment.

### Applications of patient-derived organoids

For decades, a plethora of research aimed to provide pivotal information on treatment selections, specifically for cancer patients. However, despite the extensive research in the field, only a handful of markers could be used with high predictive values for precision medicine in oncology. The mechanisms of responsiveness, adaptive and acquired resistance, and patient heterogeneity are still incompletely understood and hence treatment personalization is beyond reach. For example, preclinical and clinical research together showed that the combination of targeted therapies, like BRAF and MEK inhibition in melanoma and lung cancer, EGFR and MET inhibition in lung cancers, or BRAF and EGFR inhibition in colorectal cancer could lead to prolonged benefits in selected patient groups. The drug development process is also inefficient in the absence of predictive *in vivo* and *in vitro* models. Consequently, only a limited number of drugs passed drug approvals frequently, without biomarkers for selecting patients. For example, for colorectal cancer (CRC), only 20–30 drugs are currently approved despite more than 4000 conducted clinical trials. Similar trends can be observed for other cancers (ClinicalTrials.gov).

In this regard, recently emerged patient-derived organoid (PDO) models proved to be potentially valuable tools for drug development and as biomarkers (Fig. 3). PDOs have revolutionized biomedical research and personalized medicine by providing highly relevant models for studying human diseases. These organoids, which are cultured from patient-specific cells, closely mimic the architecture and function of the original tissues, allowing for more accurate disease modeling. In cancer research, PDOs enable the study of tumor behavior and drug response in a patient-specific context, facilitating the development of tailored therapeutic strategies.

By bridging the gap between traditional cell cultures and *in vivo* models, PDOs enhance our ability to predict clinical outcomes, thereby advancing personalized treatment approaches and contributing to more effective healthcare solutions.

#### Preclinical approaches to modelling cancer

Cancer research progression demanded the development of proper models to enable in-depth oncology studies. Thus, experimental need boosted the development of corresponding cancer models, here exemplified by colon cancer models.

The first experimental models for colon cancer were 2D cell lines and were established in the 1970–1980s (HT29, HCT116, Caco-2, SW480, LoVo) (von Kleist et al., 1975, Brattain et al., 1984). Those immortalized cell lines are derived from human adenomas or adenocarcinomas of colon cancer patients. Although simple models, they provided a huge amount of information for CRC modeling, including target identification and effective drug combinations for different sub-groups of CRC patients (Berg et al., 2017, Jaaks et al., 2022).

Given the limitations of 2D models, further research development for more representative cancer models was required. The first genetically engineered mouse model (GEMM) of colorectal cancer was introduced in 1990 – the APCMin mouse model (Min – multiple intestinal neoplasia) (Moser et al., 1990). This mouse model harbors an inactivating mutation in the APC gene and thus mimics tumor development similar to colorectal cancer patients with APC mutations (60–70 % of all CRC cases). This model has been useful for studying the initial steps of cancer development but has several limitations: 1. young age of the mice at the onset of tumor development, 2. the predominant tumorigenicity in the small intestine which typically does not progress to more advanced adenocarcinoma stages. The design and implementation of the Cre-loxP system combined with common cancer driver genes such as APC, TP53 and KRAS led to the development of more advanced mouse models (Lakso et al., 1992, Bürtin et al., 2020).

In addition to GEMMS, patient-derived xenografts (PDXs), whereas human colorectal tumor samples are implanted in immune-deficient mouse hosts, were also successfully used to model the spreading of human metastatic tumor cells after injection in mice.

Animal models have greatly advanced our understanding of the disease and despite a general trend in decreasing use of animals following the 2014 EU Directive (2010/63/EU) to ensure animals used for scientific purposes are more protected, they are still heavily used in medical research. The use of animal models such as mouse, zebrafish and Drosophila has greatly impacted our understanding of cancers and how specific mechanisms contribute to disease. However, they often lack the desired level of translational capabilities in human settings. Human diseases have a higher level of heterogeneity, and individual tumors harbor a higher level of complexity. Personalized approaches using PDOs present a much more reliable model for translational research and selection of treatment strategies.

##### Patient-derived organoids to replace animal models in preclinical studies

3D *in vitro* modeling with human cells is increasingly recognized as a valuable alternative to animal models, not only in cancer research but across various scientific fields. As our understanding of the physiological differences between animals and humans grows, there is a strong movement towards reducing reliance on animal models in preclinical studies.

PDOs present a great alternative to animal models for preclinical studies. They highly reflect the tumor cells’ behavior in comparison to generated GEMMs, therefore being a more representative model for translational research. It has been shown that PDOs recapitulate the histopathological features and mutational spectrum of original tumors and therefore provide representative models to study specific cancers, especially for precision medicine decision-making (van de Wetering et al., 2015, Roerink et al., 2018, Fujii et al., 2016, Ooft et al., 2019, Bruun et al., 2020). The establishment of PDOs is very efficient and reaches 90 % while the efficiency of PDX establishment is around 70 % (van de Wetering et al., 2015). The research utilizing PDOs unraveled a high level of inter-patient heterogeneity including morphological heterogeneity, genetic heterogeneity, and heterogeneity in terms of drug response, which may be related to the different spectra of acquired mutations for each individual tumor (van de Wetering et al., 2015, Fujii et al., 2016, Vlachogiannis et al., 2018). The application of PDOs seems to be a useful tool for predicting tumor response to standard drug treatments which would facilitate and speed up the therapy decision-making process (Vlachogiannis et al., 2018, Ooft et al., 2019, Narasimhan et al., 2020). However, it must be noted, that human organoid cultures still use animal products, such as Matrigel or animal-derived growth factors. It has been found that animal products lead to inconsistent results due to batch-to-batch variability (Hockney et al., 2023). Although there is a push towards using synthetic materials in cultures, this practice is not yet widespread. Consequently, *in vitro* culture methods are not entirely “animal-free”.

A critical advantage of organoids for clinical oncology decision-making is modeling therapy resistance and resistant clones to predict the following line of therapy for individual patients or drug combinations that would most successfully prevent resistance and treat the different clones of the same tumor. The expectation is that more personalized approaches will emerge based on PDOs soon.

### Challenges and limitations of patient-derived organoids

Despite their obvious potential as *in vitro* models for personalized medicine, drug discovery and basic research, organoids have several limitations that hinder their preclinical and clinical implementation. Although the field has seen a surge in protocols for generating organoids from various tissues and tumor types (Driehuis et al., 2020), the efficiency and reproducibility of these methods could be improved. Indeed, for a few cancer types and subtypes (e.g. prostate), the efficiency of organoid derivation and their expansion is extremely low (Gao et al., 2014). This low efficiency is sometimes detrimental due to the high costs associated with generating organoids.

At present, organoids are still of micron scale, and can only recapitulate a subset of the functions of normal tissues. The main gap resides in the lack of a vascular system and native tissue microenvironment, and incomplete ECM. When organoids grow to a certain size, the cells in the center cannot get enough nutrition and oxygen due to limited diffusion range, and excretion of metabolic waste from the cells is difficult. This limitation prevents the recapitulation of complex physiological processes, restraining the use of organoids for certain applications.

Vascular networks are key to supply nutrients, oxygen and growth factors to cells within tissues. Two main methods can be employed to construct vascularized organoids: one is *in vivo* vascularization by transplanting organoids into animal models, and the other is *in vitro* vascularization, which is implemented by combining co-culture with vascular cells and microengineering. Limited examples have shown the ability to drive organoid vascularization after *in vivo* transplantation into hosts, leading the generation of functional vascular networks. In the *in vitro* approach, endothelial cells (ECs) and other parenchymal tissue are either templated on a scaffold to form a vascular bed or self-organize within the tissue following induction with angiogenic factors (Nashimoto et al., 2017, van den Berg et al., 2018, Zhao et al., 2021).

Another main challenge in organoid culture is the inability to accurately represent the diversity of the cell types found in the tumor microenvironment (TME). The TME, which includes stromal cells and immune cells among others, is crucial for tumor growth and for therapy response. A first challenge lies in the heterogenous spatial distribution of the TME, which might result in variability in the cellular composition of resected tissues used for organoid generation (Allam et al., 2022). More importantly, established organoid cultures typically do not support the long-term co-culture of TME cell types. We have improved protocols to maintain TME cell types over time in culture. Specifically, we optimized media for melanoma organoids cultured in hydrogel microwell arrays. Using immunofluorescence (IF) and quantitative RT-PCR, we demonstrated that these organoids partially maintained MFAP5-expressing cancer-associated fibroblasts (CAFs) over multiple passages, indicating they can replicate TME heterogeneity *in vitro* (own unpublished data).

The variability in culture protocols presents another challenge in the organoid field. Non-standardized methods across different research institutions introduce technical variability, making it challenging to reproduce results and compare data across studies. This variability arises from differences in tissue sources and freshness of tissue, processing techniques, medium formulations, and the use of animal-derived 3D matrices. These matrices, such as Matrigel, are derived from mouse sarcomas and contain a complex mix of ECM proteins and growth factors. However, they suffer from batch-to-batch variability, are not able to fully replicate specific properties of the human tumor ECM and are also very costly. Despite this, researchers have compared different commercially available ECM providers and found that while different ECM sources have a significant effect on organoid growth speed in pancreatic cancer organoids, the drug response and gene expression remain consistent across multiple lots and commercial sources (Lumibao et al., 2024).

ECM is a complex hierarchical network composed of proteins, which affect a series of cell processes, such as adhesion, proliferation, and differentiation. The construction of biomaterials that mimic natural ECM characteristics is significant for studying cell physiology (Zhu et al., 2019). The development of synthetic and engineered biomaterials offers another avenue to address the limitations of current ECM components (Poudel et al., 2021). For instance, Gjorevski and colleagues introduced PEG-based matrices for intestinal organoids, showing that specific matrix properties are required to support stem cell colony formation and subsequent differentiation (Gjorevski et al., 2016). A hydrogel is a multiphase matrix composed of hydrophilic polymers with high water content, which has a highly porous structure similar to the natural ECM. Hydrogels are recognized as the first choice for simulating biological ECM to construct organoids *in vitro* because of their high biocompatibility, extraordinary permeability, appropriate elasticity, and hardness (Liu et al., 2019).

Microfluidic technologies and organoids-on-a-chip platforms offer promising solutions to integrate vascularization and dynamic environmental cues into organoid cultures. These systems can mimic blood flow and nutrient gradients, and can accommodate co-culture with immune cells, providing a more realistic tumor microenvironment (Cui et al., 2020). For instance, at Doppl, we use microfluidics to generate 3D tubular perfusable mini-intestines with user-defined crypt and villus-like domains, supporting a higher degree of cell-type diversity and enabling studies on cancer cell interactions with the ECM (Nikolaev et al., 2020).

Finally, the ethical concerns in using organoids for cancer research are complex and multilayered. One significant issue is the sourcing of tumor tissues, which requires obtaining samples from patients under informed consent, raising questions about the ethical handling of these tissues. The process of acquiring and using human tissues remains a very lengthy process and needs strict adherence to ethical guidelines and regulatory compliance to ensure that patient rights and confidentiality are protected.

Despite these challenges, significant advancements have been made, including novel protocols for maintaining all TME cell types, the development of synthetic and defined hydrogels, and innovative technologies like microfluidic devices and organoids-on-a-chip. These innovations are enhancing the reliability and applicability of organoid models, paving the way for more standardized, efficient, and ethical methods for organoid culture, which hold great promise for future clinical applications.

## Scalable organoids

### Scalable organoids for high throughput screening (HTS)

Organoid research, despite its transformative potential in modeling human tissues and diseases, faces significant challenges related to variability, reproducibility, and scalability, with this being applied to organoids derived from both PSCs and adult ASCs. Traditional organoid culture methods are labor-intensive, prone to human error, and suffer from batch-to-batch variability, which historically prevented their application in high-throughput screening (HTS) and large-scale studies.

Recent advancements in automation technology offer promising solutions to these challenges. Innovations such as robotics equipped with cooling stations and liquid handlers (allowing Matrigel and other ECM handling), microfluidics systems (Selimović et al., 2011, Piraino et al., 2012, Tu et al., 2014), advanced imaging, and image analysis tools have revolutionized how organoids are cultured and analyzed.

Over the past 10 years, several liquid handling systems were integrated into specific working systems (work cells) with automation and software solutions designed to streamline and automate organoid culture and screening. These platforms provide high precision in liquid handling and can automate organoid cell seeding, media changes, and small-volume compound addition during HTS campaigns (Louey et al., 2021, Boehnke et al., 2016).

Indeed, studies have shown that robotic systems can generate organoid arrays with success rates comparable to manual methods (close to 100 %), with enhanced efficiency and reproducibility (Brandenberg et al., 2020).

Liquid handlers can be combined with high-content and automated imaging systems (e.g. IN Cell Analyzer 2000; Operetta CLS) in air/temperature-controlled work cells. Automated imaging systems coupled with sophisticated image analysis pipelines enable high-resolution imaging and high-throughput phenotypic screening of organoid growth and morphology.

In summary, high-content organoid screening platforms integrate automated liquid handling, imaging, novel organoid culture technologies and superior image analysis to assess the effects of various bioactive compounds on both cancer and healthy organoids. These platforms can screen large libraries of drugs by generating dose–response curves and evaluating the efficacy and toxicity of compounds in a high-throughput manner.

Several studies have demonstrated the effectiveness of automation in organoid research. As an example, we described an automated workflow for fabricating mouse intestinal organoid arrays using a sophisticated robotic liquid handling system (Hamilton Microlab STAR). Briefly, organoids were manually dissociated, and the resulting cell suspension was processed by the pipetting station, which consistently seeded mouse intestinal stem cells in 96-well microarray plates followed by growth media dispensing. The performance of robotically generated organoid arrays was comparable to manually generated arrays, with no significant differences in the percentage of microwells containing stem cell colonies after 2–3 days. The screening protocol also included automated imaging and single-organoid resolution for analysis, where live and dead cells were fluorescently labeled for high-content screening (Brandenberg et al., 2020). A similar approach was established using metastatic colorectal cancer organoids (Tan et al., 2023).

The semiautomated drug assay using patient-derived tumor organoids (PDTOs) involved the standardization of a 384-well format organoid platform for high-throughput drug profiling. Similarly, The PDTOs were processed through a series of washes, enzymatic digestion, and filtration to create single-cell suspensions. Single dissociated cells were then seeded into wells using a Microfluidic Liquid Dispenser, which allows cooling of Matrigel. Drug treatments were applied using an automated liquid Handler, which allowed testing single agents and combinations in several doses. Daily imaging of the cultures was performed using an automated imaging system. With the developed semi-automated organoid workflow, drug testing has shown an 85 % accuracy rate in predicting how well patients respond to treatment.

In summary, the automation of organoid culture and screening processes is not only feasible but also essential for overcoming the current challenges of variability, reproducibility, and scalability in organoid research. Although most assay readouts are image-based, several other organoid/cell-based assays such as viability assays, flow cytometry (FACS), gene expression analysis (qPCR), proteomics, and others, can be integrated into automation platforms, enhancing the depth of biological information that can be gathered within one screening campaign and thus increasing the scope and utility of organoid studies. By integrating advanced technologies and automated workflows, we can significantly enhance the efficiency and accuracy of organoid-based studies, propelling this field toward its full potential in high-throughput applications.

Recently, organoid bioreactors were designed to provide a controlled and scalable environment for the culture of organoids (Licata et al., 2023). These bioreactors maintain optimal conditions for organoid growth, including nutrient supply, oxygen levels, and waste removal. This dynamic environment mimics the physiological conditions found *in vivo* more accurately than static cultures. Bioreactors can be equipped with sensors to monitor and adjust parameters such as pH, temperature, and oxygen concentration in real-time, further enhancing the reproducibility and scalability of organoid cultures. Microfluidics, for instance, provide a controlled environment that surpasses static cultures by ensuring consistent nutrient supply and waste removal, while embedded sensors monitor and adjust conditions in real-time.

As we look into the future, advanced and defined organoid scaffolds present a key area for development. Organoid scaffolds are designed to support the growth and organization of cells in three dimensions, mimicking the ECM found *in vivo*. These scaffolds aim to enhance the structural and functional fidelity of organoids, providing a more accurate representation of human tissues. By offering a defined environment that promotes cell–cell and cell-matrix interactions, these scaffolds pave the way for more accurate disease models and therapeutic screens.

## Scaffold-guided organoids

By carefully studying human organs and understanding embryonic development at various stages, scientists quickly linked mechanical cues, geometry, and shape to the function of specific tissues. Geometry-driven differential gene expression patterns were first observed in developing Drosophila and Xenopus embryos, where genes driving cell fate decisions are modulated through cytoskeletal re-organization within rapidly growing tissues (Martin et al., 2009).

These discoveries in developmental biology inspired mechanobiologists working with mammalian cells to pivot from two-dimensional geometry and mechanic cell perturbations to more physiological three-dimensional scaffolding methods (Engler et al., 2006).

Advanced bioengineering techniques were developed to generate 3D scaffolds with defined mechanical, chemical and geometrical properties. The mostly predominant techniques consist in: (i) locally tethering ligands in fully defined, naturally derived or decellularized matrices within which cells are embedded, (ii) bioprinting, (iii) hydrogel embossing using microfabricated molds as well as (iv) laser microfabrication.

In 2012, Gjorevski and Nelson developed a method to fabricate geometry-guided 3D epithelial tissues in collagen type I matrices and quantified the distribution of forces within this construct. They could demonstrate that the force distribution within the fabricated scaffold was significantly different between a cell collective and a single cell (Gjorevski and Nelson, 2012). This work led to the demonstration that the geometric configuration of host epithelial tissues can significantly affect the invasiveness of breast cancer cells. Understanding how specific geometric configurations can either promote or inhibit cancer cell invasion provided valuable insights for developing new therapeutic strategies targeting the physical aspects of the tumor microenvironment (Gomez et al., 2010).

While current organoid culture systems primarily rely on cell-intrinsic self-organization, the integration of external geometrical constraints as well as locally presented biochemical and physical cues is essential for creating more physiologically relevant organoids.

Wang et al demonstrated that organoid-derived adult intestinal stem cells, when seeded on “crypt-like” naturally derived extracellular matrix (ECM) surfaces, can maintain their stemness hallmarks only when residing at the bottom of the microfabricated crypts, while the most differentiated intestinal cell types were most abundant at the surface of the scaffold. They also report the need of the cells niched in the crypt-like structures to be exposed to stem-cells expansion factors on the basal side and to more differentiation factors on the apical side of the tissue construct thus mimicking the in-vivo growth factors gradients. They show that their colonic tissue construct harbors similar functions to those of the naïve intestinal epithelium (Wang et al., 2018).

Before the advent of scaffold-guided organoid development, symmetry breaking and tissue patterning of the intestinal crypt was widely attributed to random and transient expression of mechanotransduction markers like YAP, along with the subsequent activation of the Notch pathway (Serra et al., 2019). However, by using organoids shaped like their in-vivo counterparts, researchers discovered that YAP expression was deterministically confined to the crypt-like domains, driving stem cells to localize in areas that most closely resemble native tissue (Gjorevski et al., 2022).

These significant advancements in understanding how tissue shape influences cellular behavior and organoid formation accelerated the adoption of scaffold-guided organoids to engineer more complex intestinal tissues from the various segments of the gastro-intestinal tract such as the small intestine and the colon as well as of other healthy organs such as the bile duct (Nikolaev et al., 2020, Elci et al., 2024). When grown in pre-shaped scaffolds, organoids of these various tissues reorganize into structures harboring more complex architecture at the macroscopic tissue level. This higher level of complexity supports maintaining the stem and progenitor compartment while enabling the emergence of more differentiated cells not typically found in conventionally cultured organoids.

More recently, the same approach was used to understand how the tumor microenvironment complexity influences colorectal tumor growth within the healthy epithelium using autologous patient-derived primary organoids. This demonstration offers for the first time an in-vitro tool to understand drug efficacy and specificity as well as how cells of the tumor microenvironment such as the CAFs, the tumor infiltrated lymphocytes (TILs) and the vasculature modulate these responses and confer cancer cell resistance to these anticancer therapies (Lorenzo-Martín et al., 2024).

The correlation between tissue shape and function is becoming a significant research area, with several studies highlighting how geometrical and mechanical properties of tissues influence their biological behavior and functionality. The remarkable advancements in microfabrication processes and biomimetic polymer engineering enabled the field of bioengineering to guide stem and progenitor cells as well as macroscale tissue growth *in vitro*. While patterning the adult intestinal and the mammary epithelium has been widely studied, the approaches described in this section would greatly benefit other complex epithelial tissues such as the lung and the liver to improve our understanding of human tissue homeostasis as well as disease onsets *in vitro*.

## Testing immunotherapy

Cancer immunotherapy attracted its attention due to its recent advances, especially in treating melanoma patients. In the case of melanoma, checkpoint inhibitors alone or in combination increased the survival rates by 5-fold for patients with melanoma (Knight et al., 2023). To date, a few biomarkers have been suggested to identify patients with the highest chance of benefits: 1. PD-L1 expression levels, 2. tumor mutational burden, 3. microsatellite instability (MSI). Response to immunotherapy is still highly variable in cancer patients, but the presence of tumor-reactive T cells is a promising biomarker. The identification of TILs and their tumor-reactivity could be an important aspect when selecting immune therapy. The use of PDOs is particularly attractive for this purpose as tumor organoids not only harbor the relevant antigens, but also represent a heterogeneous complex system composed of different cell types with their unique transcriptional states (e.g. levels of PD-L1 expression).

We and others have shown that immunotherapy outcomes could be successfully mimicked ex-vivo using PDOs co-culture with TILs either using classical droplet-based or suspension-based methods (Dutta et al., 2021). We have performed co-culture/killing assay experiments using autologous or allogenic PDOs and TILs for multiple patients with colorectal cancer. TILs can be isolated from cancer biopsies and expanded in interleukin −2 (IL2)-conditioned media. While TILs from some patients can effectively kill autologous cancer cells, TILs from other patients are ineffective. Further experiments showed a much higher level of T cell exhaustion (PD1, TIM3, LAG3 levels) in the TILs, which were ineffective, potentially explaining the observed results. Moreover, validating the T cell states opens the possibility for fine-tuning immunotherapy, selecting combination therapies, with PDO-TILs co-cultures to improve outcomes.

## The patent landscape in organoid technology

The patent landscape in organoid technology is rapidly evolving and plays a significant role in shaping research, development, and commercialization of organoid and its applications. Many organoid-based start-ups and businesses have emerged over the past decade, creating an integrated industry that covers everything from reagents and consumables to disease models and therapeutic applications. The growing commercial value of organoid technology is also reflected in the exponential rise in number of organoid patents since 2015, with a wide range of applications across industries (Zhu et al., 2022). Organoid-related patents allow inventors, companies and research institutions to secure exclusive rights over critical technologies, methodologies, and materials used in the production, maintenance and applications of organoids. These patents often cover key processes such as cell reprogramming, differentiation protocols, and the construction of organoid models. For example, by 2022, 672 organoid-related patents could be identified with 76.64 % of patents related to model development (Zhu et al., 2022).

In general, patents give inventors the right to exclusively exploit their inventions economically for a given period and come with specific rights depending on the national patent legislation. In Europe for example, patents are regulated by the European Patent Convention (EPC) and the EU Biopatent Directive (Bartels, 2021). According to this, organoids are patentable when they fulfill the general patentability requirements of “invention”, “novelty”, “inventive step” and “susceptibility of industrial application” and their generation does not require the use of human embryos (Wolff, 2024). For example, brain organoids made from hiPSCs meet this criteria and patents and multiple patents have already been granted for methods to produce brain organoids (Wolff, 2024).

While the growing patent landscape increases the market value of organoid technologies, it also adds complexity to organoid research by controlling access to key technologies, raising concerns over high costs, and challenges in collaboration. Patents can create barriers to entry into the organoid research field, particularly for mid-sized industrial laboratories or academic institutions that may struggle with licensing fees or navigating complex patent portfolios. This restricts access to cutting-edge techniques and potentially slows down the translation of research into industrialized applications thus limiting the scope of scientific advancements (Hernández-Melchor et al., 2023). In addition, patents may restrain innovation by preventing researchers, especially in industry settings, from utilizing certain materials or methods due to concerns over intellectual property interference. This often results in fragmented research efforts, where collaboration between researchers and institutions is hindered by legal and financial concerns over intellectual property rights.

To address these challenges, the organoid research community needs to explore alternative strategies, such as revisiting patent frameworks, developing collaborative agreements or defining overarching organoid utilization guidelines for research and development that facilitate a wider access to organoid technology without slowing down innovation and translation. Balancing the need to protect intellectual property with the broader goals of scientific progress and ethical responsibility will be crucial as the organoid patent landscape continues to evolve.

## Challenges and future directions

In this review, we highlighted the advantages and the remaining limitations of both iPSC-derived and ASC/PDO-derived organoids. iPSC-derived organoids have the extraordinary capacity to give rise to all cells of the human body thanks to their pluripotent nature. However, their differentiation remains cumbersome in length, protocol intricacy, cell maturation and costs. To address these challenges, the iPSC research community built high quality and standardized biobanks of iPSC lines as well as tissue-specific progenitors as starting material for organoid generation. This enabled the generation of more reproducible and better controlled iPSC-derived organoids, notably for liver and intestinal differentiation.

On the contrary, ASC-derived organoids, including PDTOs, can be efficiently generated from primary material of individuals and can faithfully reproduce tissue-specific functions in-vitro in less than two to four weeks. However, the introduction of other organ resident tissue compartments such as neural, immune, vascular, and scaffolding cells relies on the separation of these various cell types from the primary material with single cell sorting techniques. The organ or tumor resident cells are often ill-defined, challenging to isolate and phenotypically drift when grown *in vitro* ahead of their reconstruction with the target tissue. In addition, the amount of available primary material greatly complexity this process as from one biopsy not all cell types can be obtained.

Recently established bioengineering technique have the potential to close these gaps thus enabling the various cell types to be differentiated and obtained within the final organoid construct without the need of intermediate steps that induce phenotypic and molecular aberrations.

iPSC differentiation into multi-cell type organoids using conventional self-organization have the potential to further differentiate cells of the target tissue while maintaining the stem and progenitor niche thanks to the presence of phenotypically accurate supporting cells of the tissue microenvironment. This approach is currently being explored for notoriously difficult tissues to mature form iPSCs *in vitro*. Moreover, the advent of bioengineered organoid construct guided by organ-specific geometries have the potential to standardize this process and establish even more self-sustaining and more differentiated iPSC-derived organoids. Recently, functional, and complex scaffold-guided ASC-derived organoids have been published and showed significant improvements in sensitivity to currently available therapies in the context of oncology. While this demonstration still relied in reassembling cell types that may have lost their tissue-specific phenotype and molecular hallmarks, adopting a similar approach using primary material directly may give rise to truly organ mimicking tissue constructs in the dish. In addition to reproducing the target organ in-vitro these approaches may enable the generation of tissue constructs with a complete biological complexity by, for example, adding the microbiota compartment to the epithelial barriers of the human body. These constructs will enable us to finally understand onsets of the currently epidemic yet uncurable chronic human diseases such as cancer, inflammatory bowel diseases, diabetes, and obesity.

Given these trajectories, organoids are set to gain increasing value and play a more significant role in drug development and disease research. As the field evolves, the expanding patent landscape of organoid technology is shaping both research directions and commercialization efforts, highlighting the field’s growing impact on the biomedical industry. While patents are crucial for protecting intellectual property and fostering innovation, they can also create barriers by limiting access and complicating collaboration, particularly for smaller institutions. Managing this patent complexity while aligning intellectual property rights with the broader goals of scientific advancement and ethical standards will be essential moving forward.

## Conclusions

The advent of organoid technology marks a breakpoint in biomedical research, exceeding previous limitations of scientific investigation. This advance has changed our understanding of human biology and introduced unique avenues in disease management. At the tip of this field, the possibilities seem unlimited with organoid technology poised to remodel healthcare, research methodologies, and ethical considerations concerning human life.

In this review, we have highlighted iPSC-derived organoids emphasizing their utility in studying human development, disease modeling and genome engineering. The bespoke nature of organoid systems, particularly patient-derived organoids, has introduced a new era in biomedical research. By closely mimicking the intricate architecture and functionality of human organs, organoids offer a more representative model for human physiology and pathology, outdoing traditional *in vitro* and animal models. The capacity of organoids to replicate complex biological processes, such as the tumor immune microenvironment and host-pathogen interactions, positions them at the front in further elucidating disease mechanisms and therapeutic responses. Looking to the future, the integration of organoids with cutting-edge technologies such as novel bioengineering technologies, synthetic biology, gene editing, and artificial intelligence heralds a transformative phase in biomedical innovation. This synergy holds promise for unlocking new therapeutic pathways, advancing drug development, and providing new insights into complex diseases. The potential of advanced organoids to serve as platforms for rapid drug testing and functional experimentation, coupled with their compatibility with gene-editing techniques, signifies a jump in medical research.

To conclude, organoid technology stands at the forefront of a revolution in the biomedical field. Its impact on human society will be profound, and its future applications appear limitless. As researchers and clinicians continue to exploit the power of this technology, we anticipate a future full of cutting-edge discoveries and innovative treatments that will ultimately remodel our approach to understanding and curing human diseases.

## Declaration of competing interest

The authors declare the following financial interests/personal relationships which may be considered as potential competing interests: The Ecole Polytechnique Fédérale de Lausanne has filed for patent protection on one of the technologies described herein, and N.B is named as an inventor on this patent. N.B. is shareholder in Doppl SA, which is commercializing this patent. E.H., F.P., M.C., A.R. and N.B. are employees of Doppl SA.

## Data Availability

No data was used for the research described in the article.
